# Bioelectrical impedance vector analysis (BIVA) in university athletes

**DOI:** 10.1186/s12970-020-00403-3

**Published:** 2021-01-09

**Authors:** Priscila Custódio Martins, Luis Alberto Gobbo, Diego Augusto Santos Silva

**Affiliations:** 1 Departamento de Educação Física, Universidade Federal de Santa Catarina, Centro de Desportos, Campus Universitário – Trindade – Caixa Postal 476, Florianópolis, Santa Catarina CEP 88040-900 Brazil; 2 Universidade Estadual Paulista, São Paulo, Brazil

**Keywords:** Body water, Body composition, Athletic performance, Sports

## Abstract

**Background:**

Bioelectrical impedance vector analysis (BIVA) is able to identify differences in hydration status and body composition components, such as body cell mass (BCM) by means of plotting individuals in ellipses, when comparing groups with different characteristics.

**Objective:**

Compare the confidence and tolerance ellipses of BIVA in individual and team sports athletes with a non-athlete reference population.

**Design and participants:**

One hundred sixty-seven college athletes (team sports: 117 athletes, individual sports: 50 athletes) aged between 18 and 35 years. Bioelectrical impedance was used to measure resistance (R) and reactance (Xc) values that were used in the BIVA analysis to identify hydration status and BCM, respectively. Hotelling’s t-test was used to identify differences between groups in the confidence ellipses and the comparison was made with a non-athlete Italian reference population.

**Results:**

There were no differences between male team sports and individual athletes (*p* = 0.151) and for female athletes (*p* = 0.624). Most athletes were located in the 50% tolerance ellipses, indicating adequate hydration. Compared to the Italian a non-athlete population, athletes of both sexes presented left impedance vector deviation in the minor axis (Xc) of the tolerance ellipses, indicating higher BCM.

**Conclusion:**

There were no differences in BIVA between team and individual sports athletes, but most athletes presented an adequate hydration state and, compared to a non-athlete population, the athletes of the present study presented higher BCM.

## Introduction

Bioelectrical impedance vector analysis (BIVA) derived from resistance and reactance measurements is a method used to identify nutritional status and to monitor hydration status in different populations [1–3]. The BIVA is able to identify differences in the hydration status in which the resistance/height axis (long vector) is observed and in the components of body composition, such as body cell mass (BCM), where the reactance/height axis is observed (short vector) through the graph of individuals in ellipses, when comparing groups with different characteristics [4–6].

In the sporting context, different studies have been carried out to identify the athlete’s hydration status, as this information is important for analyzing sports performance [6–8]. In addition, monitoring the hydration status and body fluids can help to identify athletes at higher risk of injuries due to the state of dehydration and assist in prescribing fluid intake [4, 6, 9, 10] The identification of injuries and follow-up during recovery until the return to activity depends on expensive methods, which are not always accessible to all clubs and institutions, such as creatine kinase analysis and magnetic resonance imaging. Therefore, BIVA research in the field of sports medicine is justified because the method evaluates in real time, non-invasively and with relatively low cost [4].

BIVA also allows estimation BCM. Studies have made comparisons between athletes and non-athletes and identified that athletes presented BIVA shift to the left in the reactance axis, indicating higher BCM when compared to non-athlete population [10, 11]. Other studies performed comparisons among athletes of different performance levels and observed that when compared to amateur athletes, professional athletes presented higher BCM [7, 12, 13]. In addition, research investigated athletes in different periods of the season and found that BCM reduced after sports competition compared to the pre-competition period [14].

Studies have observed that, after sports competition, there are changes in the ellipses of tolerance of BIVA, with vector shortening [15], or even declining vector to the left [7]. These changes can indicate hyperhydration and adequate hydration of athlete, respectively [16]. Other studies have interpreted the BIVA in order to identify the amount of BCM [6, 7], which can also be obtained by through estimates made with the BIA technique.

In this sense, the importance of assessing body composition, especially BCM, is highlighted, since physical stress caused by the training load and participation in sports competitions can cause changes in BCM that can be harmful to physical performance and cellular health of athletes [11, 17]. In addition, body composition has been used to identify athletes of different performance levels and has been shown to have an impact on sports success [5]. Although different studies have performed comparisons between athletes and non-athletes [10, 11], no study have compared team and individual sports athletes, which requires further investigations, since body composition and physical training are different according to the sport specificities [18], which may lead to differences in BIVA analysis.

The literature reports that different intensities and volumes of training have a different impact on body composition, muscle strength and other physical parameters. Thus, it is important to compare different sports. A study identified that individual sports had a higher volume and training load when compared to team sports [18]. In addition, athletes practicing individual sports participated in more sports competitions [18]. A study carried out with athletes of different sports modalities found higher phase angle values in weight lifters, judo and gymnastics athletes (individual sports), when compared to athletes practicing soccer and hockey (team sports) [17]. A study carried out with university athletes of collective sports (basketball, volleyball, soccer and hockey) and individual (rowing and gymnastics) observed that the athletes of rowing and gymnastics, that is, individual sports, have lower values of body fat when compared to sports athletes collective. On the other hand, athletes who practice team sports have higher values of fat-free mass [19]. On the other hand, athletes of team sports presented higher impedance values [17]. These findings reinforce the need to investigate other fluid distribution and cellular health indicators, such as BIVA in team and individual sports athletes. In this way, the aim of this study was to compare the BIVA confidence and tolerance ellipses between university team and individual sports athletes and a non-athlete population.

The hypotheses of the present study are that individual sports athletes present higher BCM concentrations when compared to team sports athletes because the training load and volume are higher in athletes who practice individual sports compared to those practicing team sports which may favor BCM gains and that when compared to a non-athlete population, athletes present different BIVA vector position indicative of body composition adaptations due to differences in training and performance [12].

## Method

### Study design

This cross-sectional study is part of the macroproject entitled “Effects of a sports season on the cellular health of athletes of different modalities” carried out at the Federal University of Santa Catarina (UFSC), located in the city of Florianópolis, Santa Catarina, Brazil. Data collection was carried out between the months of September and November 2017. The research was approved by the Ethics Committee for Research with Human Beings at UFSC, under the number: 2.308.476. All university athletes signed the Free and Informed Consent Form.

### Participants

The population was composed of university athletes from different sports, regularly enrolled in Undergraduate and Graduate Studies at UFSC. The study sample was convenience, so that all athletes who participated in training for sports competitions, aged 18 to 35 years old, of both sexes, were recruited. The total number of athletes linked to the sports modalities was 179, according to the most important university competition in the state.

The inclusion criteria were: 1) athletes aged 18 to 35 years age, of both sexes; 2) regularly enrolled at UFSC; 3) participate in at least one state, regional, sports competition.

national and / or international throughout a sporting season in the year 2017. The exclusion criteria were: 1) athletes who were submitted surgeries that alter body composition, such as bariatric surgery; 2) trained athletes, or those who no longer had a relationship with UFSC. Athletes who rejected the invitation were considered as refusals to participate in the study during the data collection period. The athletes who agreed to participate in the study, but did not attend the meeting data collection, up to three attempts were considered as losses. Thus, six athletes were considered refusals, and six were considered as losses. Thus, 167 athletes were evaluated, 92 were male [athletics (*n* = 11), badminton (*n* = 02), basketball (*n* = 01), field football (*n* = 30), futsal (*n* = 14), hockey grass (*n* = 03), judo (*n* = 08), swimming (*n* = 07), skateboard (*n* = 01), tennis (*n* = 4) and volleyball (*n* = 11)], and 75 females [athletics (*n* = 06), badminton (*n* = 01), flag football (*n* = 13), futsal (*n* = 20), handball (*n* = 09), grass hockey (*n* = 04), judo (*n* = 06), skateboard (*n* = 03), tennis table (*n* = 01) and volleyball (*n* = 12)].

**Fig. 1:**
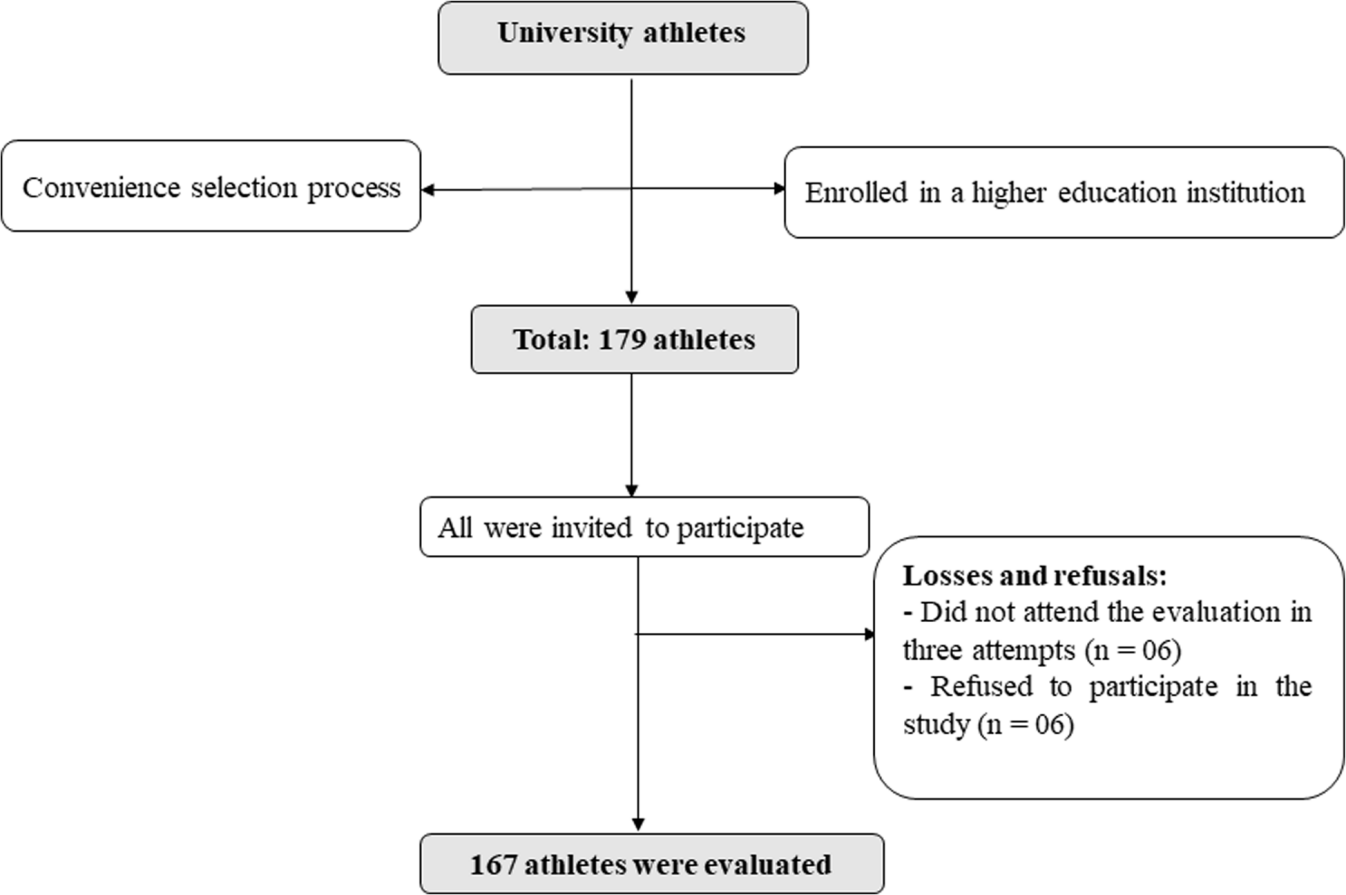
Flowchart of the study sampling process

The modality practiced was categorized into team sports (basketball, flag football, field soccer, futsal, handball, field hockey and volleyball), and individual and / or double sports (track and fields, badminton, judo, swimming, skateboarding and table tennis). Team sports are characterized by intermittent loads, with the aerobic system predominating [20]. On the other hand, individual or collective sports mainly use the anaerobic system, which can influence the shape and body size characteristics [12]. In addition, the loss of body water can be accentuated in individual sports in which sports are divided into weight classes, such as judo [21]. The practice time, the number of sports competitions per year and the presence of injuries were obtained through the application of an anamnesis form, answered individually by each athlete (Fig.  1).

### Bioelectrical impedance vector analysis (BIVA)

In order to evaluate BIVA, the bioelectrical impedance (BIA) method was used. The model used was InBody® 720 (Biospace, Los Angeles, USA), multi-frequency with eight electrodes, measuring impedance in five frequencies (1, 50, 250, 500 and 1000 kHz) and Xc in three (5, 50 and 250 kHz). The technique provided impedance and reactance data at frequency of 50 kHz, calculating the resistance value, and then the phase angle using the arc tangent formula (Xc / R) × 180 ° / π^3^, with values expressed in degrees. The BIA model used presented acceptable reproducibility and accuracy level for the estimation of body composition tissues at frequency of 50 kHz, when compared to plethysmography and dual X-ray absorptiometry [22]. In addition, the manufacturer reinforces that high accuracy level is obtained by following the correct measurement procedures.

Based on BIVA values, confidence and tolerance ellipses were created. Confidence ellipses comprise the 95% confidence interval for the vector means found by plotting the mean of components of the relationship between R and Xc by height (meters) measured in a group of individuals [23]. The sample mean is presented as an estimate of results that would be obtained if the total population was studied. Confidence intervals are used to verify whether a mean is significantly different from a hypothetical value or a comparison population [23]. Tolerance ellipses are graphic analyses of individual or the three ellipses: the median, the third quartile and the 95th percentile, which are regions that include 50, 75 and 95% of individual points, respectively [23]. Thus, the tolerance graph allows a more detailed classification of the vector position of the individual impedance (one point) in the R / Xc chart by means of its distance from the mean vector of the reference population [23].

For BIA evaluation, height was measured by the protocol of the International Society for the Advancement of Kinanthropometry (ISAK) by level-1 researcher using AlturaExata® stadiometer (Belo Horizonte, Brazil), with 1-mm resolution. During evaluation, athletes remained in orthostatic position, holding two levers and with their feet positioned on a platform. The evaluation lasted about 2 min. All athletes were instructed to follow pre-test recommendations, which included: fasting for at least 4 h, light clothing (bikini, swimwear, top, lycra shorts), barefoot, without the use of earrings and / or rings and / or any kind of metal, abstaining from intense physical activity the previous day, abstaining from drinks with high caffeine content in the previous 12 h. Female athletes who were in the menstrual period on the day of the evaluation were rescheduled for another time. The evaluations took place in the morning, between 8 a.m. and 12 p.m.

### Statistical methods

For the descriptive analysis of data, mean and standard deviation were calculated. Kurtosis and asymmetry were used to verify data normality (interval between − 2 and + 2). The Student’s T-test was used to identify differences between team and individual sports athletes. For confidence ellipses, R and Xc values were standardized by height (meters) and differences between groups were analyzed by the T^2^ Hotelling test. The T^2^ Hotelling test was designed to compare population mean vectors. For tolerance ellipses, R and Xc values standardized by height for all sample and the means standardized in z-score were used. All analyses were performed using BIVA 2002® software (Microsoft, Padova, Italy), adopting *p* ≤ 0.05.

## Results

Study participants were 167 team and individual sports athletes (male = 92; female = 75). The mean age of males was 22.18 (± 3.47) years and females was 22.98 (± 3.25) years. Male athletes practicing team sports presented higher mean R and Xc values when compared to individual sports athletes (*p* < 0.01). For female athletes, only R value was higher in athletes practicing team sports compared to individual sports athletes (*p* < 0.01). Male athletes practicing individual sports participated in more competitions when compared to team sports athletes (*p* < 0.01). Athletes of both sexes of individual sports had higher weekly training load volume (*p* < 0.01) (Table 1).

**Table 1:** Characteristics of the sample stratified by gender and practiced modality

	**Male (*n*=92)**			**Cohen’D**	**Female (*n*=75)**			
	**Team Sports (*n*=59)**	**Individual Sports** **(*n*=33)**	***p*-value**		**Team Sports (*n*=58) (*n*=58)**	**Individual Sports (*n*=17)**	***p*-value**	**Cohen’D**
	**Mean (±sd)**	**Mean (±sd)**			**Mean (±sd)**	**Mean (±sd)**		
**Body mass (kg)**	72.14 ±9.08	71.72 ±8.46	0.82	0.04	59.99 ±8.19	59.00 ±9.09	0.66	0.11
**Height (cm)**	178.48 ±5.86	176.28 ±6.95	0.11	0.48	165.18 ±6.29	162.10 ±6.31	0.08	0.34
**R (Ω)**	470.44 ±44.10	445.14 ±50.34	**0.01**	0.54	579.33 ±59.36	547.05 ±52.17	**0.04**	0.55
**Xc (Ω)**	53.60 ±5.63	50.37 ±6.96	**0.01**	0.52	54.96 ±6.03	53.16 ±5.07	0.26	0.30
**R/H (Ω/m)**	264.68 ±26.05	253.52 ±31.68	0.31	0.38	350.54 ±40.98	340.07 ±34.03	0.92	0.27
**Xc/H (Ω/m)**	30.22 ±3.61	28.60 ±4.34	0.66	0.54	33.39 ±4.47	32.76 ±3.46	0.79	0.15
**Practice time (months)**	8.81 ±11.00	12.84 ±15.00	0.14	0.32	9.94 ±13.12	7.76 ±11.22	0.53	0.17
**Competitions**	1.30 ±1.23	3.11 ±3.51	**<0.01**	1.37	1.78 ±1.55	1.69 ±1.81	0.83	0.05
Training load (frequency * min)	239.27 ±90.83	337.77 ±101.08	**<0.01**	1.04	224.48 ±99.12	334.50 ±93.49	**<0.01**	1.12

*n* sample, *sd* standard deviation, *kg* kilograms, *cm* centimeters, *m* meters, *min* minutes, *R* resistance, *Xc* reactance, H: height

**Fig. 2:**
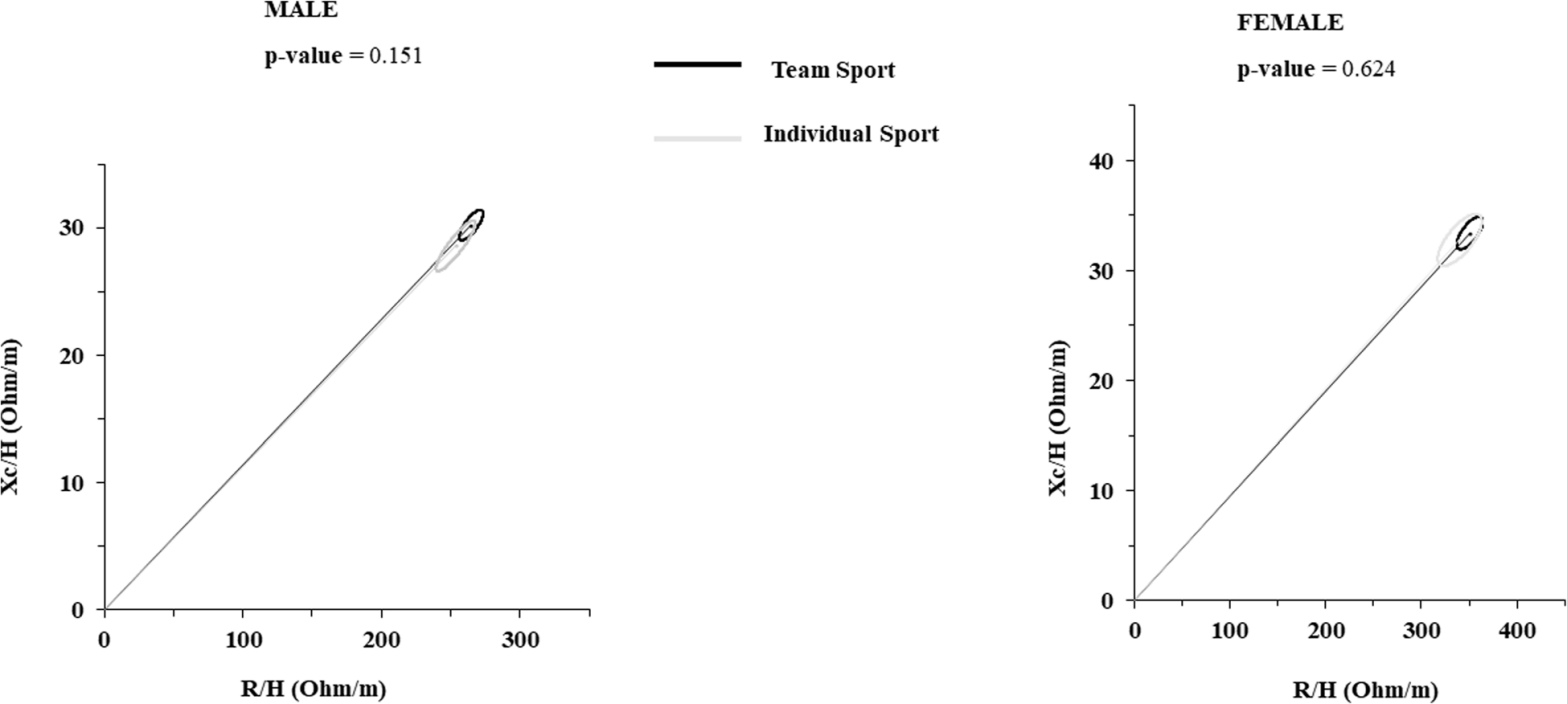
Mean impedance vectors with 95% confidence ellipses of athletes practicing team sports in comparison with the ellipses of athletes practicing individual sports

BIVA confidence analyses demonstrated that ellipses were overlapped, demonstrating that there were no differences between individual and team sports athletes. T^2^ Hotelling test confirmed that for male athletes (*p* = 0.151) and for female athletes (*p* = 0.624), no difference between team and individual sports was observed (Figs. 1 and 2).

**Fig. 3:**
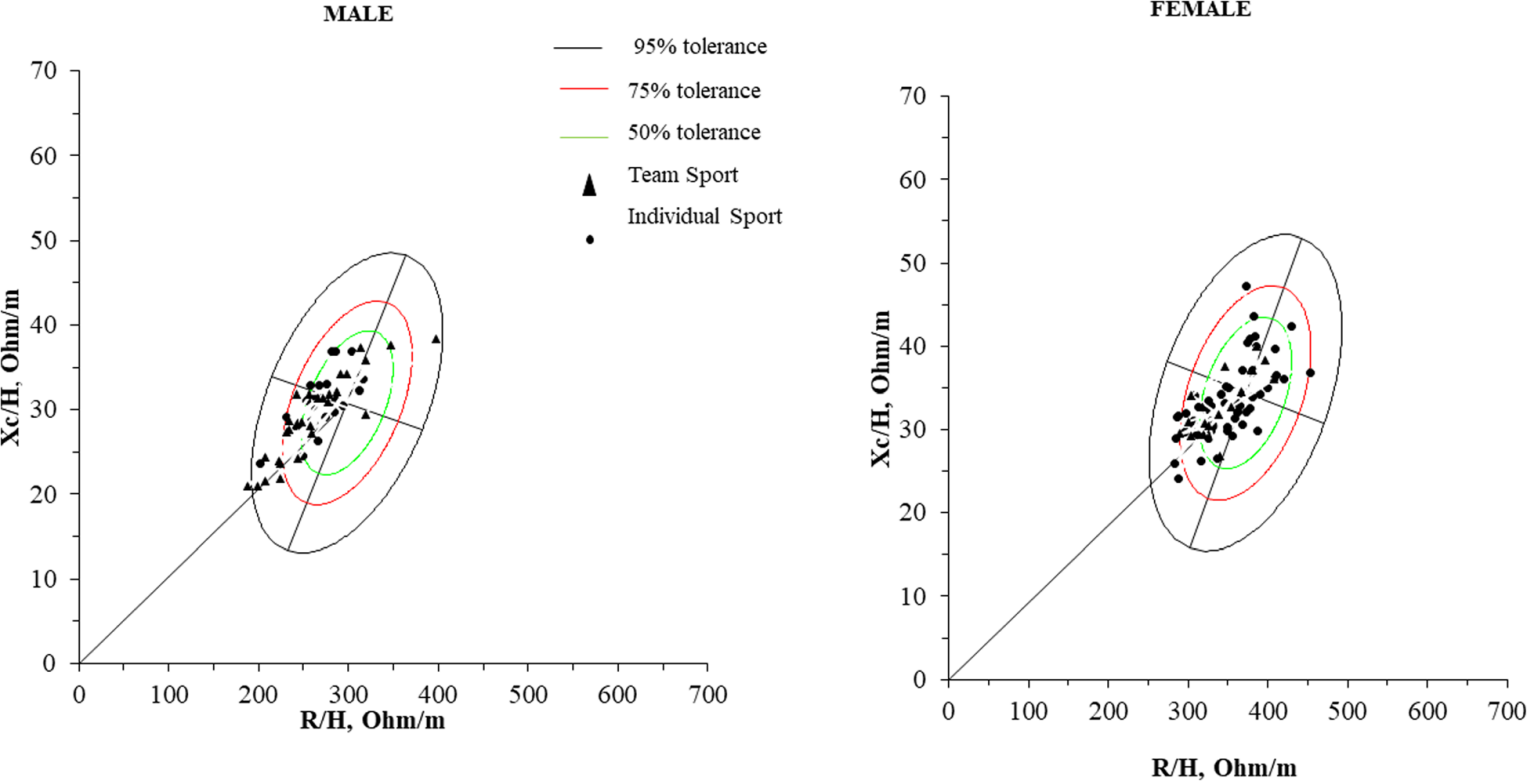
Mean impedance vectors with tolerance ellipses of 50%, 75$ and 95% of athletes practicing team sports in comparison to the ellipses of athletes praticing individual sports

Figure 3 shows the vectors of all athletes in relation to tolerance ellipses of 50, 75 and 95% with the healthy Italian reference population. Most male athletes of both modalities were located in 50% tolerance ellipses, indicating normal hydration compared to the Italian population. Compared with the healthy Italian male population, male athletes of both individual and team sports showed impedance vector shifted to the left on the minor axis (Xc) of tolerance ellipses, indicating higher BCM. Most female athletes were located in the 50% tolerance ellipses, with impedance vector shifted to the left on the minor axis (Xc) of the tolerance ellipses, as compared to the healthy Italian female population and non-athletes, indicating higher BCM.

## Discussion

The main results of the present study were: 1) in confidence and tolerance ellipses, no differences between team sports athletes and individual sports athletes were observed; 2) the majority of male and female athletes practicing team and individual sports were located in the 50% tolerance ellipses, indicating adequate hydration and; 3) in comparison with the Italian non-athlete population, athletes of both sexes showed impedance vector shift to the left on the minor axis (Xc) of the tolerance ellipses, indicating higher BCM.

When analyzing confidence and tolerance ellipses, no differences between athletes practicing team and individual sports were observed in the present study. A study conducted with Italian volleyball athletes identified differences between elite and low-performance athletes [11]. Although the training load and the number of sports competitions were higher in individual sports, BIVA analysis showed no differences, which may suggest that in addition to aspects related to physical training, other factors may also impact BIVA, such as body mass and height, which would not differ in athletes investigated in the present study.

The results of the present study indicated that the majority of male and female team and individual sports athletes were located in the 50% tolerance ellipses, indicating adequate hydration. Similar results were observed in studies with athletes of different sports and levels [5, 7, 10, 11]. Micheli et al. [7] using BIVA in a cross-section design found a decrease in the R/height component and an increase in phase angle without differences in the Xc/height component in elite soccer players compared to lower performance level athletes [7]. A study carried out with 27 judo athletes during a sports season, identified that, regardless of body composition changes, athletes who increase Xc and R reduced extracellular water and body fluids while those who raised phase angle increased intracellular water. Judo’s athletes who reduced weight, decreased fat-free mass but cellular health was not compromised, as phase angle remained stable and, consequently, cell hydration [24]. These findings reinforce the use of BIVA in the sports context to monitor athletes’ hydration status.

In the present study, individual and team sports athletes of both sexes showed impedance vector shift to the left on the minor axis (Xc) of the tolerance ellipses, in comparison with the Italian non-athlete population. These results indicate an increase in BCM, which may reflect specific adaptations of physical training in the athletes’ body composition [12]. A study with 525 male athletes of different performance levels found that athletes had higher BCM when compared to non-athletes [5]. A study with female volleyball players found no differences between sexes when comparing BCM [8]. Therefore, it is speculated that the sport adaptations are similar to male athletes because differences in body composition decrease when individuals are trained, especially in athletes who practice the same sport [20]. Piccoli et al. [25] compared the BIA measures of bodybuilders with a non-athlete and found reductions in the average resistance / height component of BIVA and small differences in the average reactance / height component. The authors observed that both components of the vector decreased in bodybuilders compared to non-athletes, but with a higher rate of reactance: resistance (i.e., a greater phase angle). This result indicates that the electric current flowed through more body fluids with more cells per unit volume (volume of intracellular fluid).

This study presented results for male and female athletes, which allowed identifying possible differences between sexes. Another strong point of this study is the BIVA investigation in athletes, a subject still little studied in the area of body composition that, for a long time was only concerned with body fat and lean mass distribution.

This study also presents limitations such as the use of a reference Italian population; however, no studies that proposed cut-points for the adult non-athlete population in Brazil were found. The lack of information regarding the reliability of the BIA device used can be considered a limitation of the study. The absence of information on body perimeters made it impossible to use specific BIVA, considered more robust for the analysis of body composition in athletes [4]. However, a study identified that, specific BIVA turns out to be more accurate for the analysis of % fat mass in athletes, while it does not correctly evaluate TBW, for which classic BIVA appears to be a suitable approach. Phase angles, and hence both BIVA approaches, can detect proportion of extracellular and intracellular water changes [26].

In addition, the 4-h fast of food and liquids for the assessment of BIA can be considered a limitation of the study. Although the standard protocol for the BIA test suggests an 8-h fast, Androutsos et al. [27] recently reported that the intake of food and liquids impacted approximately 1% on the components of body composition, when the fast was only 2 h. Thus, other studies with athletes used periods of less than 8 h of fasting for evaluations, due to the characteristics of the investigated sample, such as the large volume of training and the proximity to sports competitions [28]. Another limitation of this research was not carrying out the test of urine specific gravity [29] The urine specific gravity is the biochemical marker most commonly used in research and applied settings to detect water deficits in athletes [29]. However, there is a discussion on the accuracy of this technique to identify the hydration status, and some studies have found inconsistency between different techniques [30, 31]. Thus, the use of BIVA can be a non-invasive strategy to identify the athlete’s hydration status.

### Practical applications

From the results found, coaches can use BIVA to monitor hydration status and BCM loss throughout the sports season, in team and individual sports athletes. For the analysis, coaches will only have to apply an BIA assessment, an easy-to-handle method that can be taken to the sporting environment with practicality.

## Conclusions

It was concluded that there were no differences in BIVA analysis between athletes practicing team and individual sports, but most athletes presented adequate hydration status and in comparison to the Italian non-athlete population, athletes of the present study presented higher BCM. Thus, BIVA seems to be a promising and useful technique for the analysis of the hydration status and body cell mass in university athletes.

## Abbreviations

BIVA: Bioelectrical impedance vector analysis
BIA: Bioelectrical impedance
BCM: Body cell mass
R: Resistance
Xc: Reactance
ISAK: International Society for the Advancement of Kinanthropometry
